# Potentiating Gilteritinib Efficacy Using Nanocomplexation with a Hyaluronic Acid–Epigallocatechin Gallate Conjugate

**DOI:** 10.3390/polym16020225

**Published:** 2024-01-12

**Authors:** Ki Hyun Bae, Fritz Lai, Qingfeng Chen, Motoichi Kurisawa

**Affiliations:** 1 Bioprocessing Technology Institute (BTI), Agency for Science, Technology and Research (A*STAR), 20 Biopolis Way, Centros #06-01, Singapore 138668, Singapore; khbae@bti.a-star.edu.sg; 2 Institute of Bioengineering and Bioimaging (IBB), Agency for Science, Technology and Research (A*STAR), 31 Biopolis Way, The Nanos #08-01, Singapore 138669, Singapore; 3 Institute of Molecular and Cell Biology (IMCB), Agency for Science, Technology and Research (A*STAR), 61 Biopolis Drive, The Proteos, Singapore 138673, Singapore; sclai@imcb.a-star.edu.sg (F.L.); qchen@imcb.a-star.edu.sg (Q.C.); 4 Key Laboratory for Major Obstetric Diseases of Guangdong Province, The Third Affiliated Hospital of Guangzhou Medical University, Guangzhou 510150, China; 5 Graduate School of Advanced Science and Technology, Japan Advanced Institute of Science and Technology, 1-1 Asahidai, Nomi 923-1292, Ishikawa, Japan

**Keywords:** hyaluronic acid, gilteritinib, epigallocatechin gallate, nanocomplex, leukemia

## Abstract

Acute myeloid leukemia carrying FMS-like tyrosine kinase receptor-3 (FLT3) mutations is a fatal blood cancer with a poor prognosis. Although the FLT3 inhibitor gilteritinib has recently been approved, it still suffers from limited efficacy and relatively high nonresponse rates. In this study, we report the potentiation of gilteritinib efficacy using nanocomplexation with a hyaluronic acid–epigallocatechin gallate conjugate. The self-assembly, colloidal stability, and gilteritinib loading capacity of the nanocomplex were characterized by reversed-phase high-performance liquid chromatography and dynamic light scattering technique. Flow cytometric analysis revealed that the nanocomplex efficiently internalized into FLT3-mutated leukemic cells via specific interactions between the surface-exposed hyaluronic acid and CD44 receptor overexpressed on the cells. Moreover, this nanocomplex was found to induce an eradication of the leukemic cells in a synergistic manner by elevating the levels of reactive oxygen species and caspase-3/7 activities more effectively than free gilteritinib. This study may provide a useful strategy to design nanomedicines capable of augmenting the therapeutic efficacy of FLT3 inhibitors for effective leukemia therapy.

## 1. Introduction

Acute myeloid leukemia (AML) is a devastating hematopoietic malignancy with an overall 5-year survival rate of only 25% [1]. Over the past decades, FMS-like tyrosine kinase receptor-3 (FLT3) mutations have been the subject of intensive research because they are the most common type of genetic alterations observed in AML (20–30% of patients), and are associated with increased relapse and poor survival [2]. Although several FLT3 inhibitors have been discovered and tested in clinical trials, gilteritinib (GLT) is currently the only medication approved by the U.S. Food and Drug Administration (FDA) as a monotherapy for the treatment of AML patients harboring FLT3 mutations [3]. In the randomized phase III trial, GLT treatment greatly extended the median overall survival (9.3 months) compared to conventional salvage chemotherapy (5.6 months) in patients with refractory/relapsed FLT3-mutated AML [4]; however, nearly one-fourth of GLT-treated patients showed no response, and only 20.6% of them survived for more than 2 years, highlighting the unmet clinical need [5]. In this perspective, it is desirable to develop advanced nanoparticle formulations capable of enhancing the anti-leukemic activity of GLT for more effective AML treatment.

Hyaluronic acid (HA) is a linear and non-sulfated carbohydrate polymer that consists of disaccharide repeating units of *N*-acetyl-_D_-glucosamine and _D_-glucuronic acid [6]. Owing to its unique viscoelastic, non-immunogenic, and biodegradable properties, HA has been widely explored for diverse biomedical and pharmaceutical applications, such as viscosupplementation, drug delivery, soft tissue augmentation, and cartilage tissue engineering [7,8,9]. Interestingly, several attempts have been made to utilize HA for targeted delivery of anti-leukemic drugs because of its high specific affinity towards the cluster determinant 44 (CD44) receptor overexpressed on AML cells [10]. For example, Qiu et al. designed CD44-targeted HA-mercaptopurine prodrug via a glutathione-sensitive carbonyl vinyl sulfide linker and described its enhanced accumulation and greater cytotoxicity on leukemia cells than free mercaptopurine [11]. Moreover, Sun et al. reported that the conjugation of HA on curcumin-loaded liposomes endowed them with a high CD44-binding affinity (*K*_d_ = 49.95 pM) and more potent therapeutic efficacy in the xenogeneic AML mouse model when compared with nontargeted liposomes and free curcumin [12].

**Figure 1:**
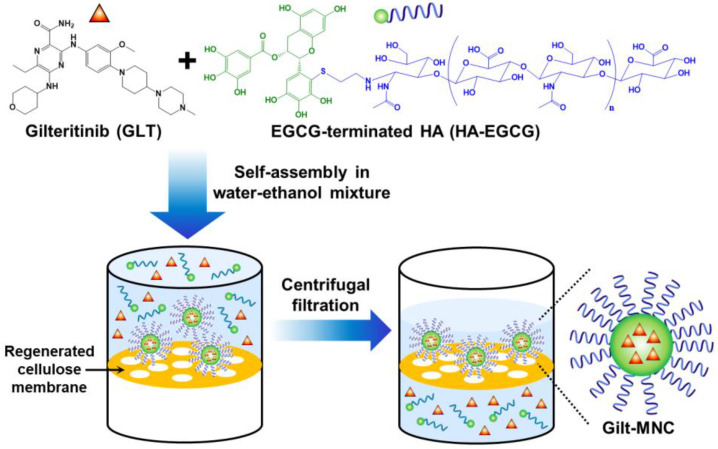
Schematic illustration of the self-assembly of Gilt-MNC and subsequent purification using centrifugal filtration through a regenerated cellulose membrane. Reprinted/adapted with permission from Ref. [16]. Copyright 2022, the Authors.

Epigallocatechin gallate (EGCG) is the most prevalent green tea catechin and has received growing attention as a promising adjuvant for leukemia therapy [13]. Multiple studies have reported that EGCG differentially triggers apoptosis in leukemic blast cells but not in normal blood cells [14,15,16]. In addition, EGCG has been documented to potentiate the therapeutic efficacy of conventional anti-leukemic drugs, such as As_2_O_3_, 5-aza-2′-deoxycytidine, and all-*trans* retinoic acid by elevating the intracellular levels of reactive oxygen species (ROS), including hydroxyl radical (·OH) and peroxynitrite (ONOO^−^) [17,18,19]. In this study, we report the construction of a gilteritinib-loaded micellar nanocomplex (Gilt-MNC) via the co-assembly of a GLT and HA-EGCG conjugate in water–ethanol mixture and subsequent centrifugal filtration (Figure 1). Gilt-MNC was designed to efficiently internalize into leukemic blast cells via specific interactions between the surface-exposed HA and CD44 receptor overexpressed on the cells. This proof-of-concept study verified the capability of Gilt-MNC to induce a synergistic eradication of FLT3-mutated AML cells through enhanced ROS production and caspase activation compared to free GLT.

## 2. Materials and Methods

### 2.1. Materials

HA (*M*_w_ = 20 kDa) was a product of Lifecore Biomedical (Chaska, MN, USA). Gilteritinib (GLT) was acquired from MedChemExpress (South Brunswick, NJ, USA). EGCG was a product of DSM Nutritional Products Ltd. (Heerlen, The Netherlands). Amicon Ultra-15 centrifugal filter devices, NaCl, and urea were obtained from Merck Millipore Corporation (Darmstadt, Germany). Triton X-100, Tween 20, bovine serum albumin (BSA), 4′,6-diamidino-2-phenylindole (DAPI), and dextran from *Leuconostoc* spp. (*M*_w_ = 15–25 kDa) were obtained from Sigma-Aldrich (St. Loius, MN, USA). RPMI 1640 media, DMEM media, penicillin/streptomycin solution, fetal bovine serum (FBS), phosphate-buffered saline (10 mM, pH 7.4, PBS), DyLight 488 maleimide, CellROX Green oxidative stress detection reagent, and Image-iT LIVE Red apoptosis detection kit were obtained from Thermo Fisher Scientific (Waltham, MA, USA). Caspase-Glo 3/7 assay kit and CellTiter-Glo viability assay reagent were obtained from Promega (Madison, WI, USA). Glass-bottom microwell dishes (dish size = 35 mm, thickness = 0.13–0.16 mm) were obtained from MatTek Corporation (Ashland, OR, USA). Float-A-Lyzer tubing (*M*_w_ cutoff = 3.5–5 kDa) was a product of Spectrum Laboratories (Piscataway, NJ, USA).

### 2.2. Preparation of Gilt-MNC

EGCG-terminated HA (HA-EGCG) was prepared by chemically conjugating thiol-terminated HA with EGCG, as reported previously [20]. HA-EGCG and GLT were separately dissolved in deionized water and ethanol, respectively. To produce Gilt-MNC, 0.5 mL of GLT solution (final concentration: 0.3–0.6 mg/mL) was added into 0.5 mL of HA-EGCG solution (final concentration: 4–10 mg/mL). After incubation at 37 °C for 2 d in the dark while shaking, the mixture was placed in a regenerated cellulose membrane (Amicon Ultra-15, *M*_w_ cutoff = 50 kDa) and then subjected to centrifugal filtration at 2000× *g* at 20 °C for 4 min. The retrieved MNC was purified through three cycles of resuspension in deionized water and centrifugation. The resulting MNC was kept at 4 °C in the dark before use.

### 2.3. Reversed-Phase High-Performance Liquid Chromatography (RP-HPLC)

To quantify the amount of GLT encapsulated within Gilt-MNC, an aliquot (100 µL) of each sample was mixed with methanol (400 µL), and then vortexed for 1 h to extract the drug from Gilt-MNC. After centrifugation at 10,000× *g* at 4 °C for 8 min, the Acrodisc mini-spike GHP filter was used to filter the supernatant. The filtered solution (20 µL) was then injected into the Discovery HS C18 column (5 µm, 4.6 mm i.d. × 250 mm, Supelco) installed on the Waters 2695 HPLC module. The mobile phase consisting of 1% acetic acid plus methanol (45:55, *v*/*v*) was delivered at a constant flow rate (1 mL/min) at 25 °C. The RP-HPLC chromatogram at 314 nm and UV absorption spectrum were acquired by the Empower 3 chromatography data software (version 7.2, Waters Corporation, Milford, MA, USA). A series of GLT solutions at varying concentrations (0.78–12.5 µg mL^−1^) was used to establish the calibration curve (Figure S1a,b). An aliquot (500 µL) of Gilt-MNC was lyophilized and weighed to measure its dry mass. The loading efficiency and drug content were calculated by the following equations:

Loading Efficiency (%) = (Mass of GLT in MNC)/(Feed mass of GLT) × 100(1)

Drug content (%) = (Mass of GLT in MNC)/(Total mass of MNC) × 100(2)

### 2.4. Physicochemical Characterization

The effective diameter and surface charge of Gilt-MNC were assessed with dynamic light scattering (DLS) technique using the NanoBrook Omni (Brookhaven Instruments, Holtsville, NY, USA). All measurements were performed at 25 °C in quintuplicate. The critical micelle concentration (CMC) of Gilt-MNC and an empty MNC (composed of HA-EGCG alone) was assessed by examining the light scattering intensity as a function of HA-EGCG concentration [21]. To understand which interactions occurred in Gilt-MNC, its effective diameter was measured in the presence of NaCl, urea, Triton X-100, and Tween 20 at varying concentrations (0.1–100 mM). To investigate the serum stability of Gilt-MNC, each sample was incubated in PBS (pH 7.4) supplemented with 10% (*v*/*v*) FBS at 25 °C. At selected time points, an aliquot was transferred to a UV-grade polymethylmethacrylate (PMMA) cuvette and analyzed using DLS to monitor the time-course change in effective diameters and zeta potential values. For drug release tests, Gilt-MNC (GLT amount = 100 µg) was placed in each Float-A-Lyzer tubing (*M*_w_ cutoff = 3.5–5 kDa) containing 6 mL of PBS (pH 7.4) without or with 10% (*v*/*v*) FBS, and then incubated on an orbital shaker at 25 °C. At indicated time points, the release fraction (100 µL) was collected and analyzed using RP-HPLC as described above.

### 2.5. DyLight 488 Tagging of Gilt-MNC

DyLight 488-tagged HA was synthesized by chemically conjugating DyLight 488 maleimide to thiol-terminated HA, according to our previous report [20]. HA-EGCG and GLT were separately dissolved in deionized water and ethanol, respectively. To produce DyLight 488-labeled Gilt-MNC, 0.5 mL of GLT solution (final concentration: 0.4 mg/mL) was added into a mixture (0.5 mL) of DyLight 488-labeled HA and HA-EGCG (1:4 weight ratio, final concentration = 8 mg/mL). After incubation for 2 d at 37 °C in a dark place while shaking, the mixture was placed in a regenerated cellulose membrane (Amicon Ultra-15, *M*_w_ cutoff = 50 kDa) and then subjected to centrifugal filtration at 2000× *g* at 20 °C for 4 min. The retrieved MNC was purified through three cycles of resuspension in deionized water and centrifugation. The resulting MNC was kept at 4 °C in the dark before use.

### 2.6. Flow Cytometry Analysis

The human acute monocytic leukemia MOLM-14 and MV-4-11 cell lines were purchased from the American Type Culture Collection (ATCC, Manassas, VA, USA) and maintained in RPMI 1640 media containing 1% (*v*/*v*) penicillin/streptomycin and 10% (*v*/*v*) FBS. The adenovirus-transformed human embryonic kidney 293T cell line (ATCC, Manassas, VA, USA) was cultured in DMEM media containing 1% (*v*/*v*) penicillin/streptomycin and 10% (*v*/*v*) FBS. Human bone marrow stromal (HBMS) cells were obtained from Stemcell Technologies (Vancouver, BC, Canada) and cultured in complete MesenCult Proliferation Medium (Stemcell Technologies, Vancouver, BC, Canada). To examine the extent of CD44 expression, 3 × 10^5^ cells were subjected to DAPI staining and FITC anti-human CD44 antibody labeling (BioLegend, San Diego, CA, USA, clone BJ18) at 4 °C for 1 h. After rinsing using ice-cold PBS supplemented with 0.1% (*w*/*v*) BSA 3 times, the cells were analyzed using an LSRFortessa X20 flow cytometer (BD Biosciences, San Antonio, TX, USA). To verify the CD44-mediated endocytosis of Gilt-MNC, the cells were placed on 6-well plates at a density of 2 × 10^5^ cells/well and then pre-treated for 1 h with serum-free RPMI media containing either dextran or HA at a concentration of 10 mg/mL [22]. The cells were then incubated with DyLight 488-labeled Gilt-MNC dispersed in 10% FBS-supplemented RPMI media (final GLT concentration = 800 nM). After incubation for 4 h, the fluorescence intensity of the cells was examined on an LSRFortessa X20 flow cytometer (BD Biosciences, San Antonio, TX, USA).

### 2.7. Evaluation of Anti-Leukemic Activity

After seeding on white-bottomed 96-well plates at a density of 10^4^ cells/well, MOLM-14 and MV-4-11 cells were incubated in 10% FBS-supplemented RPMI media (100 µL) containing varying concentrations of Gilt-MNC, free GLT, HA-EGCG, or EGCG for 72 h. To ascertain the extent of cell survival, CellTiter-Glo assay reagent (100 µL) was added to each well before shaking for 2 min at 25 °C on a microplate shaker. After dark adaptation at 25 °C for 10 min, the luminescence signal was measured with an exposure time of 1000 ms on an Infinite 200 multimode microplate reader (Tecan Group, Mannendorf, Switzerland). Data were expressed as percentages of the relative light unit (RLU) of analyzed cells compared to RLU of the untreated cells. Half-maximal inhibitory concentration (IC_50_) of Gilt-MNC and free GLT was determined by fitting the data to a nonlinear regression model using the Prism 9 software (version 9.4.1, GraphPad Software, Boston, MA, USA).

### 2.8. Assessment of Combination Effects

The synergistic effect between GLT and HA-EGCG was investigated using the Chou and Talalay method [23]. In brief, the cell survival data were typed in the CompuSyn software (version 1.0, ComboSyn Inc., Paramus, NJ, USA), which employs the following linearized median-effect equation to create median-effect plots:

log(f_a_/f_u_) = m log(D) − m log (D_m_), (3)

where D is the dose given to the cells, D_m_ is the median effective dose, f_u_ is the fraction of live cells, and f_a_ is the fraction of dead cells. Based on the parallelism of the median-effect plots constructed, the effects of GLT and HA-EGCG were found to be mutually non-exclusive (i.e., they have different modes of action) [24]. Accordingly, the following equation for two mutually non-exclusive drugs was used to calculate the combination index (CI):

CI = (D)_1_/(ED_x_)_1_ + (D)_2_/(ED_x_)_2_ + (D)_1_(D)_2_/(ED_x_)_1_(ED_x_)_2_, (4)

where (ED_x_)_1_ and (ED_x_)_2_ are the doses of free GLT or HA-EGCG that cause x% cytotoxic effect, whilst (D)_1_ and (D)_2_ are the doses of GLT and HA-EGCG in combination required to exert the identical effect, respectively. CI > 1, CI = 1, or CI < 1 represents antagonism, summation, or synergism, respectively.

### 2.9. Measurement of Cellular ROS Production

The level of cellular ROS production was measured using the CellROX Green fluorescent sensor. Briefly, after seeding on 24-well plates at a density of 5 × 10^4^ cells/well, MOLM-14 and MV-4-11 cells were incubated in 10% FBS-supplemented RPMI media (1 mL) containing Gilt-MNC (800 nM) or free GLT at the same dose for 24 h. The CellROX Green reagent was then added to each well at a final concentration of 5 μM. After staining for 30 min at 37 °C, the cells were harvested in Eppendorf tubes and then centrifuged for 10 min at 8000 rpm at 4 °C. After discarding the supernatant, the cell pellet was rinsed with 1 mL of PBS (pH 7.4) twice to remove excess of the CellROX Green reagent. The resulting cells were dispersed in 100 µL of PBS (pH 7.4) and then transferred to each well of black-walled 96-well plates. The fluorescence intensity was recorded at an excitation wavelength of 485 nm and emission wavelength of 525 nm on a Tecan Infinite 200 multimode microplate reader. The cellular fluorescence images were obtained using an IX83 inverted microscope equipped with cellSens Dimension software (version 1.14, Olympus, Tokyo, Japan).

### 2.10. Visualization of Cellular Apoptosis

After seeding on 24-well plates at a density of 5 × 10^4^ cells/well, MOLM-14 and MV-4-11 cells were incubated in 10% FBS-supplemented RPMI media (1 mL) containing Gilt-MNC (800 nM) or free GLT at the same dose for 24 h. The cellular apoptosis was visualized using the Image-iT LIVE Red caspase-3/7 detection kit. Briefly, the cells were harvested in Eppendorf tubes and then centrifuged at 4 °C for 10 min at 8000 rpm. After discarding the supernatant, the cell pellet was suspended in 200 µL of 1X FLICA reagent working solution and then incubated in the dark for 1 h. The cells were centrifuged, washed with 1 mL of 1X wash buffer twice, and then fixed for 5 min in 100 µL of 1X apoptosis fixative solution. The cell suspension (80 µL) was applied to glass-bottom microwell dishes and then observed under an Olympus IX83 inverted microscope.

### 2.11. Quantification of Caspase-3/7 Activity

The caspase-3/7 activity of MOLM-14 and MV-4-11 cells was evaluated using the Caspase-Glo 3/7 assay kit, as reported previously [25]. In brief, after seeding on white-bottomed 96-well plates at a density of 1 × 10^4^ cells/well, MOLM-14 and MV-4-11 cells were incubated in 10% FBS-supplemented RPMI media (100 µL) containing Gilt-MNC (800 nM) or free GLT at the same dose for 24 h. Subsequently, a multi-channel pipette was used to add the Caspase-Glo 3/7 assay reagent (100 µL) to each well. After incubation in the dark for 1 h at 25 °C, the luminescence signal was acquired with an exposure time of 1000 ms on a Tecan Infinite 200 multimode microplate reader. Data were presented as percentages of the relative light unit (RLU) of analyzed cells compared to RLU of the untreated cells.

### 2.12. Statistical Analysis Methods

All the data in the current study are presented as mean ± standard deviation (SD). Statistical comparisons among three or more groups were performed using analysis of variance (ANOVA) with Tukey’s post hoc multiple comparison test, using Prism 9 software (GraphPad Software, San Diego, CA, USA). The *p* values smaller than 0.05 were considered to indicate statistically significant differences.

## 3. Results and Discussion

### 3.1. Self-Assembly and Purification of Gilt-MNC

A schematic for the self-assembly process of Gilt-MNC is depicted in Figure 1. After GLT was dissolved in ethanol, this solution was mixed with EGCG-terminated HA (HA-EGCG) dissolved in deionized water. The mixing ratio of ethanol to water was 1:1 and this condition was chosen to ensure complete solubilization of both GLT and HA-EGCG. During incubation in the water–ethanol mixture, GLT and HA-EGCG spontaneously self-assembled into nanosized Gilt-MNC having a GLT-loaded EGCG-rich core surrounded by a hydrated HA shell. Since the multiple phenolic groups of EGCG can form diverse noncovalent interactions, such as hydrophobic, H-bonding, and ionic interactions [26,27], the EGCG-rich core of Gilt-MNC was expected to act as a reservoir for efficient encapsulation of GLT. On the other hand, the surface-exposed HA chain acts as a targeting ligand for CD44 receptors that are highly expressed on leukemic cells, thus enabling AML-selective delivery of GLT [10]. Following the self-assembly process, Gilt-MNC was purified via centrifugal filtration through a regenerated cellulose membrane with a *M*_w_ cutoff of 50 kDa, which allows for efficient drainage of ethanol as well as unloaded GLT (552.7 Da) and HA-EGCG (~20.5 kDa).

**Figure 2:**
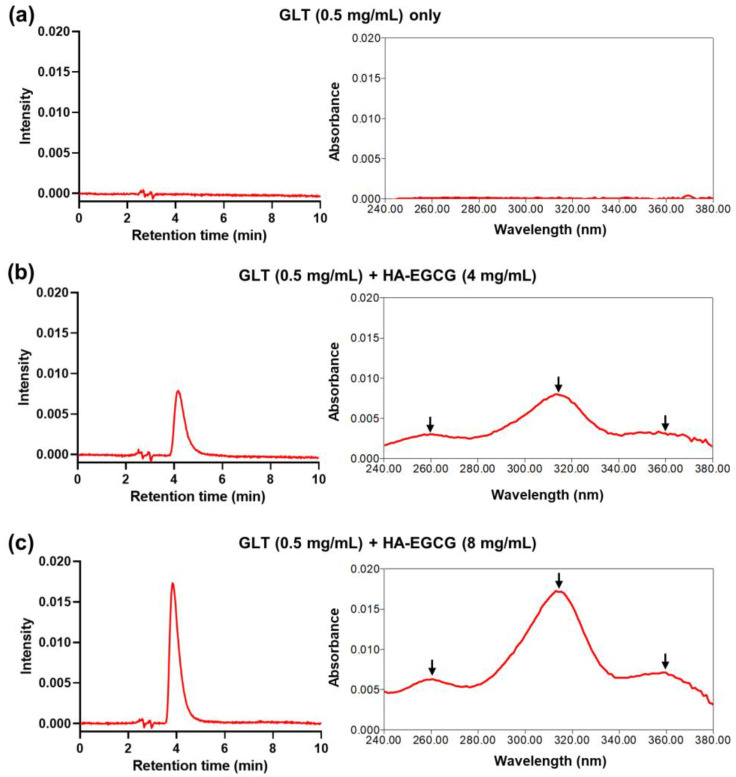
Representative RP-HPLC chromatogram (left panel) and UV absorption spectrum (right panel) of the centrifugal filtration product obtained with (**a**) GLT (0.5 mg/mL) only, (**b**) GLT (0.5 mg/mL) + HA-EGCG (4 mg/mL), or (**c**) GLT (0.5 mg/mL) + HA-EGCG (8 mg/mL). The arrows indicate the characteristic peaks of GLT at 260, 314, and 358 nm.

To verify the encapsulation of GLT within Gilt-MNC, the centrifugal filtration product was subjected to methanol extraction and subsequent RP-HPLC analysis. As shown in Figure 2a, no obvious peaks were observed in the centrifugal filtration product obtained with GLT (0.5 mg/mL) only, confirming that free GLT molecules were completely removed by the centrifugal filtration process. In contrast, when HA-EGCG was added at a concentration of 4 mg/mL, a single elution peak of GLT appeared at ~4 min in the RP-HPLC chromatogram (Figure 2b). Additionally, the UV absorption spectrum of the elution peak showed three characteristic peaks at 260, 314, and 358 nm, which were identical to those of native GLT (Figure S1c), providing evidence of the attractive interactions between GLT and HA-EGCG under this condition. A further increase in HA-EGCG concentration to 8 mg/mL resulted in a higher elution peak in the RP-HPLC chromatogram, suggesting that the intermolecular interactions were augmented at higher concentrations (Figure 2c).

### 3.2. Physicochemical Characterization of Gilt-MNC

**Figure 3:**
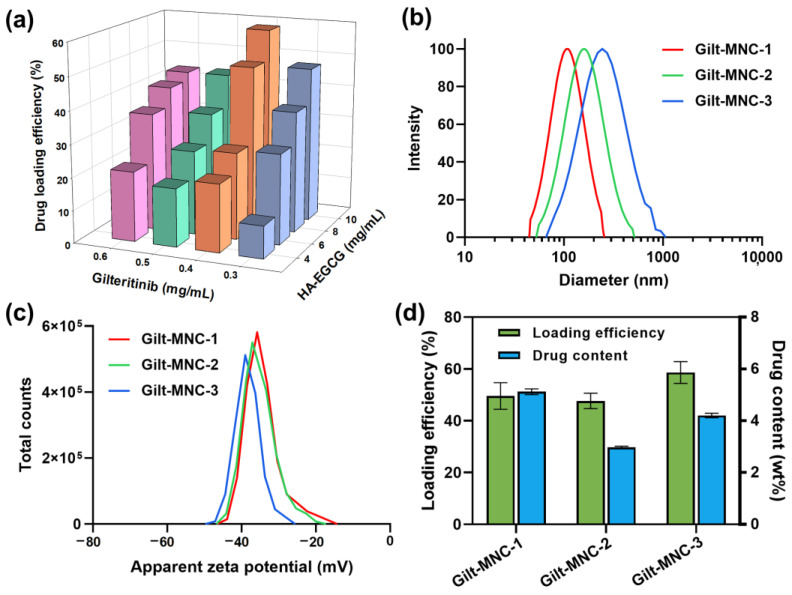
(**a**) Three-dimensional graph showing the drug loading efficiencies of a series of Gilt-MNC formed at various final feeding concentrations of HA-EGCG and GLT. The bar graphs are presented as mean values (*n* = 3). (**b**) Intensity-weighted size distribution profile, and (**c**) apparent zeta potential of Gilt-MNC-1, -2, and -3. (**d**) Comparison of the drug loading efficiencies and drug content values of Gilt-MNC-1, -2, and -3. Mean ± SD (*n* = 3).

We examined the drug loading efficiency of a series of Gilt-MNC formed at various final feeding concentrations of GLT and HA-EGCG (Figure 3a). In general, raising HA-EGCG concentrations led to a gradual increase in the drug loading efficiency, suggesting that the enhanced attractive interactions occurred at higher concentrations of HA-EGCG. On the basis of the drug loading capability, the top three formulations named Gilt-MNC-1 to -3 were screened for further investigation (Table 1). DLS analysis revealed that Gilt-MNC-1, -2, and -3 had different effective diameters of ca. 110.8, 148.6, and 176.1 nm, respectively (Figure 3b). These nanometer dimensions are desirable for intracellular drug delivery and systemic circulation [28,29]. All the formulations had highly negative zeta potential values (−34.1 to −38.2 mV), which reflects the presence of anionic HA chains on the surface of Gilt-MNC (Figure 3c). As presented in Figure 3d, Gilt-MNC-1 had a higher drug content (~5.1 wt%) and smaller effective diameter than Gilt-MNC-2 and -3, implying the formation of a more condensed micellar structure within Gilt-MNC-1. Based on the molecular weight of GLT (552.7 Da) and HA-EGCG (~20.5 kDa), it was estimated that approximately 2 GLT molecules were bound per molecule of HA-EGCG inside Gilt-MNC-1. Given the smallest particle size and highest drug content, Gilt-MNC-1 was chosen for the rest of the characterization studies.

**Figure 4:**
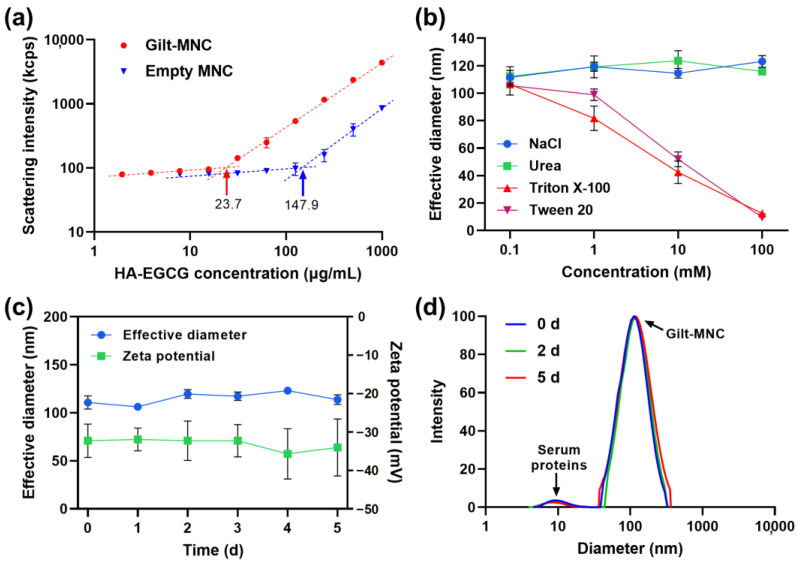
(**a**) Light scattering intensity of Gilt-MNC and an empty MNC as a function of HA-EGCG concentration. The arrows indicate the critical micelle concentration (CMC) of Gilt-MNC and an empty MNC. (**b**) Effective diameter of Gilt-MNC treated with various concentrations of NaCl, urea, Triton X-100, and Tween 20. (**c**) Time-course change in the effective diameter and zeta potential of Gilt-MNC in PBS containing 10% FBS. Mean ± SD (*n* = 3). (**d**) Intensity-weighted size distribution of Gilt-MNC in PBS containing 10% FBS measured over 5 days. The small peak of ~9.6 nm originated from the presence of serum proteins.

Next, the critical micelle concentration (CMC) of Gilt-MNC and an empty MNC composed of HA-EGCG alone was determined by examining the light scattering intensity as a function of HA-EGCG concentration [21]. The intensity of scattered light detected for Gilt-MNC began to increase linearly with HA-EGCG concentration above the CMC of 23.7 μg/mL as a result of micellar assembly (Figure 4a). Of note, the CMC of Gilt-MNC was about 6.2-fold lower than the CMC of an empty MNC (147.9 μg/mL), suggesting that the micellar structure of Gilt-MNC was more resistant to disassembly upon dilution than those of an empty MNC [30]. To understand which interactions occurred in Gilt-MNC, we monitored their particle size changes upon exposure to NaCl (an ionic bond-breaker), urea (a hydrogen bond-breaker), and Triton X-100 and Tween 20 (detergents). The addition of Triton X-100 and Tween 20 caused a marked disintegration of Gilt-MNC, whereas NaCl and urea had little influence on its particle size (Figure 4b). This finding revealed that the structure of Gilt-MNC was primarily maintained by the hydrophobic interactions between GLT and EGCG moiety, rather than H-bonding and ionic interactions. The serum stability of Gilt-MNC was examined by monitoring its particle size and zeta potential in PBS (pH 7.4) supplemented with 10% (*v*/*v*) FBS. No obvious changes in the effective diameter, zeta potential, and particle size distribution were observed for 5 days, indicating the high colloidal stability of Gilt-MNC in its physiological condition (Figure 4c,d). There was only a marginal level (<8%) of GLT leakage from Gilt-MNC in FBS-supplemented PBS over 30 h, implying that the GLT-EGCG interactions were well maintained in the presence of serum (Figure S2).

### 3.3. CD44-Targeting and Anti-Leukemic Effects of Gilt-MNC

**Figure 5:**
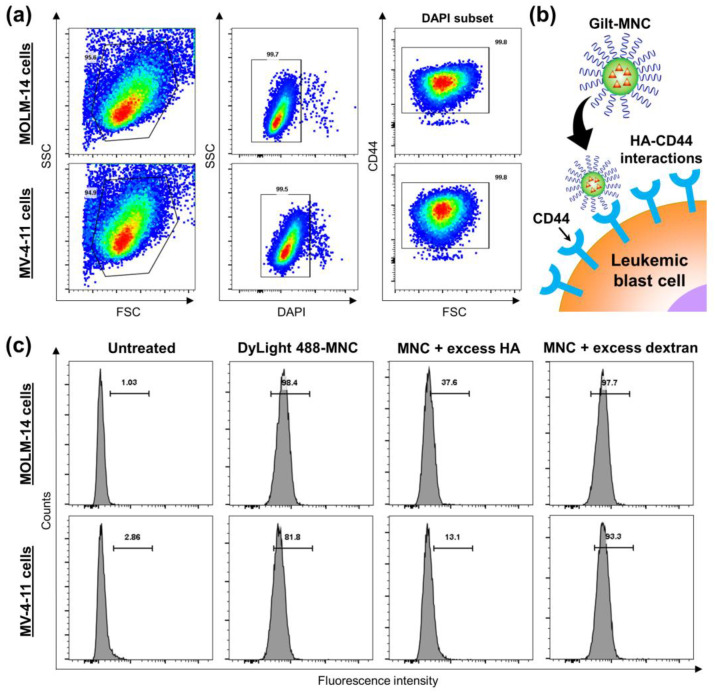
(**a**) Gating strategy used for the evaluation of CD44 expression on MOLM-14 and MV-4-11 cell lines. (**b**) Schematic illustration of the internalization of Gilt-MNC into a leukemic blast cell via HA-CD44 interactions. (**c**) Flow cytometry histograms showing the cellular accumulation of DyLight488-labeled Gilt-MNC following the treatment for 4 h with or without excess HA or dextran.

We evaluated the CD44-targeting ability of Gilt-MNC on two different AML cell lines harboring FLT3 mutations—MOLM-14 and MV-4-11. Flow cytometric analysis confirmed the abundance of CD44 expression (99.8% in DAPI subset) on both MOLM-14 and MV-4-11 cells (Figure 5a). Considering the high CD44-binding affinity of HA, it was anticipated that Gilt-MNC would efficiently internalize into these AML cells via CD44-mediated endocytosis and subsequently trigger enhanced apoptosis through the combined anti-leukemic effect of GLT plus EGCG (Figure 5b). To validate the CD44-mediated endocytosis of Gilt-MNC, the cells were pre-treated with excess amounts of free HA (CD44-blocking polymer) or dextran (non-CD44-binding polymer), followed by incubation with DyLight 488-labeled Gilt-MNC. As depicted in Figure 5c, the cellular accumulation of DyLight 488-labeled Gilt-MNC was greatly abolished by the pre-treatment of free HA, whilst no such decrease was detected after the pre-treatment of free dextran. This result proved that HA-CD44 interactions play an important role in the receptor-mediated endocytosis of Gilt-MNC into the leukemic blast cells. This CD44-targeting property of Gilt-MNC would allow for more efficient delivery of its drug payloads into CD44-overexpressing AML cells, thus helping to achieve enhanced therapeutic efficacy at lower doses.

To examine the uptake of Gilt-MNC in non-AML cells, we conducted flow cytometric analysis using two different normal cells—CD44-negative 293T cells and CD44-positive HBMS cells. As expected, negligible levels (<2%) of cellular uptake were detected in 293T cells, revealing that Gilt-MNC minimally entered normal cells lacking CD44 (Figure S3). Interestingly, HBMS cells showed much lower levels (~23%) of cellular uptake than AML cells (>80%), although they had comparable CD44 expression. It has been reported that cancerous cell membranes typically have a higher fluidity than their normal counterparts, rendering them more permeable to exogenous nanoparticles [31,32]. In this perspective, we speculate that the marginal uptake of Gilt-MNC in non-AML cells might be at least partly attributed to the inherent difference between leukemia and normal cells in terms of membrane fluidity and material transport behavior.

**Figure 6:**
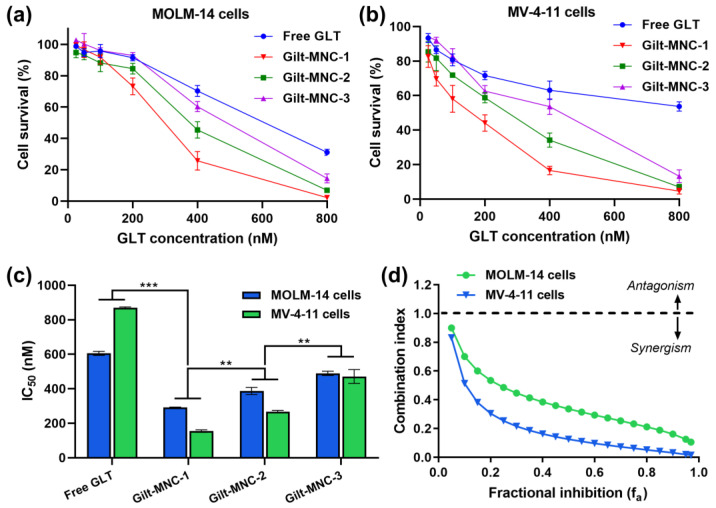
Viability of (**a**) MOLM-14 and (**b**) MV-4-11 cell lines following treatment for 3 d at varying concentrations of free GLT and Gilt-MNC. (**c**) IC_50_ values of Gilt-MNC-1 to -3 and free GLT. Mean ± SD (*n* = 3); ** *p* < 0.01; *** *p* < 0.001. (**d**) Combination index of Gilt-MNC plotted against the fractional inhibition (f_a_).

As shown in Figure 6a, all the Gilt-MNC formulations were able to eradicate MOLM-14 cells more effectively than free GLT at equivalent dosages. For instance, Gilt-MNC-1 killed nearly 74% of MOLM-14 cells at a dose of 400 nM but only a slight cell death (~30%) was induced by the same dose of free GLT. A similar trend of cytotoxicity was observed for MV-4-11 cells (Figure 6b). The quantification of the half-maximal inhibitory concentration (IC_50_) revealed that the order of anti-leukemic effect was Gilt-MNC-1 > Gilt-MNC-2 > Gilt-MNC-3 (Figure 6c). Intriguingly, this order was oppositely correlated with the effective diameters (Gilt-MNC-1: 110.8 nm < Gilt-MNC-2: 148.6 nm < Gilt-MNC-3: 176.1 nm; Table 1). Thus it was conceivable that the greatest cytotoxic effect of Gilt-MNC-1 might be attributable to its smallest particle size, which would be advantageous for intracellular uptake into the leukemic cells [28,29]. Gilt-MNC-1 was much more effective in killing the leukemic cells than free HA-EGCG when compared to equivalent EGCG unit concentrations, suggesting that the combination of GLT and HA-EGCG induced synergistic cytotoxic effects (Figure S4). To understand the combinational effects of GLT and HA-EGCG, we determined the combination index (CI) using the Chou and Talalay method [23]. The median-effect plots (Figure S5) showed that the regression lines obtained for free GLT, HA-EGCG, and their combination (Gilt-MNC) were not in parallel, indicating that GLT and HA-EGCG were mutually non-exclusive, i.e., they have different modes of action [24]. Importantly, the CI values of Gilt-MNC were smaller than 1 at all the tested levels of fractional inhibition (f_a_), representing a synergism for MOLM-14 and MV-4-11 cells (Figure 6d). These results demonstrate that the synergistic anti-leukemic effect of GLT plus HA-EGCG was responsible for the observed superior efficacy of Gilt-MNC over free GLT.

### 3.4. Evaluation of Cellular ROS Levels and Caspase-3/7 Activity

**Figure 7:**
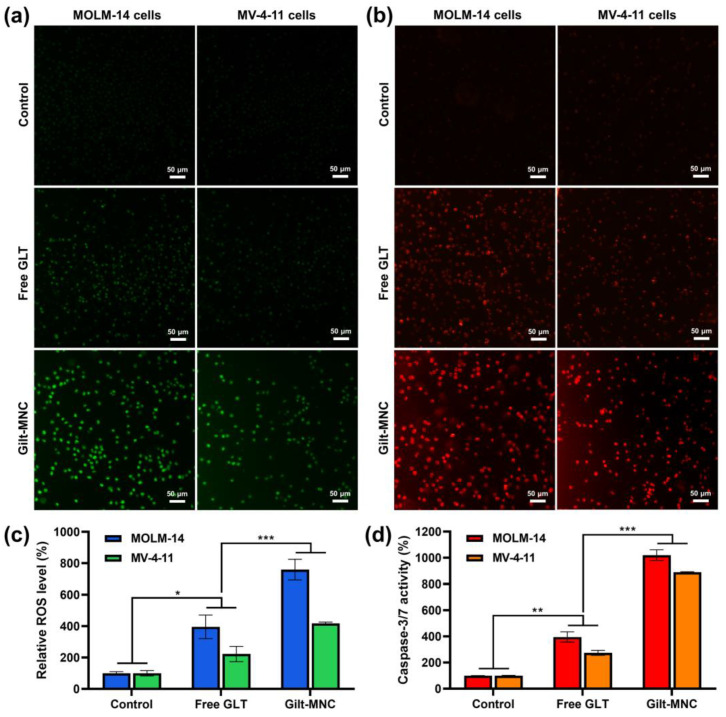
(**a**) Representative CellROX Green staining and (**b**) Image-iT LIVE Red staining images of MOLM-14 and MV-4-11 cells taken after treatment for 24 h with Gilt-MNC or free GLT at an equivalent dose of 800 nM. Scale bar = 50 μm. (**c**) Relative ROS levels detected in MOLM-14 and MV-4-11 cells subjected to the same treatments as those in (**a**). (**d**) Caspase-3/7 activities detected in MOLM-14 and MV-4-11 cells following 24 h treatment of Gilt-MNC or free GLT. Mean ± SD (*n* = 3); * *p* < 0.05; ** *p* < 0.01; *** *p* < 0.001.

Multiple studies have documented that the ability of EGCG to potentiate the efficacy of conventional anti-leukemic drugs has been associated with its pro-apoptotic effect through the overproduction of intracellular ROS [17,18,19]. In this regard, we sought to explore the effect of Gilt-MNC and free GLT on the cellular ROS levels using the CellROX Green oxidative stress sensor. As presented in Figure 7a, treatment of free GLT increased the green fluorescence in MOLM-14 and MV-4-11 cells, representing elevated ROS levels in those cells. This finding was in accordance with the previous literature reporting ROS generation in FLT3-mutated AML cells upon exposure to GLT [33]. Notably, the cells treated with Gilt-MNC displayed markedly brighter fluorescence than those treated with free GLT. Quantification of the fluorescence intensity proved that Gilt-MNC stimulated significantly greater (*p* < 0.001) ROS production than free GLT (Figure 7c).

Overproduction of cellular ROS has been reported to promote the loss of the mitochondrial transmembrane potential and cytosolic leakage of cytochrome C, ultimately leading to caspase-dependent apoptosis pathways [13]. The Image-iT LIVE Red (a fluorogenic apoptosis probe) was used to visualize the enzymatic activity of caspase-3/7 playing a central role in the caspase cascade of apoptosis [34]. We observed dramatically enhanced red fluorescence from the cells treated with Gilt-MNC (800 nM) when compared to those treated with free GLT at the same dose (Figure 7b). The caspase-3/7 activity of MOLM-14 cells substantially jumped to ~1020% upon exposure to Gilt-MNC for 24 h, while only a moderate increase (~396%) was detected from the cells treated with free GLT (Figure 7d). A similar trend of caspase-3/7 activation was also seen for MV-4-11 cells. This finding was in good agreement with the earlier ROS level analysis, implying that the pro-oxidant effects of Gilt-MNC played a major role in triggering the caspase-induced apoptotic cell death. Collectively, the above results demonstrate that Gilt-MNC effectively induced apoptosis of FLT3-mutated AML cells by facilitating CD44-targeted co-delivery of GLT and HA-EGCG as well as by stimulating ROS generation and cellular caspase-3/7 activity.

## 4. Conclusions

Nanosized Gilt-MNC was successfully constructed with the self-assembly process of GLT and HA-EGCG in a water–ethanol mixture and purified using centrifugal filtration through a regenerated cellulose membrane. The produced Gilt-MNC had a small particle size (effective diameter < 200 nm), highly negative zeta potential (−34.1 to −38.2 mV), relatively low CMC (23.7 μg/mL), and good colloidal stability in the presence of serum. Flow cytometry revealed that Gilt-MNC could be efficiently taken up by both MOLM-14 and MV-4-11 cells via CD44 receptor-mediated endocytosis. The median-effect plot analysis demonstrated that Gilt-MNC synergistically augmented the cytotoxic efficacy of GLT via the combinational delivery of HA-EGCG plus GLT. The superior anti-leukemic effect of Gilt-MNC was associated with the induction of enhanced apoptosis via elevated ROS generation and caspase-3/7 activation. Our study suggests that Gilt-MNC holds great potential as a nanomedicine for the effective treatment of FLT3-mutated AML.

## Figures and Tables

**Table 1:** Composition, effective diameter, and zeta potential of Gilt-MNC selected for characterization.

Sample Code	Final Feeding Concentration of HA-EGCG (mg/mL)	Final Feeding Concentration of GLT (mg/mL)	Effective Diameter (nm)	Zeta Potential (mV)
Gilt-MNC-1	8	0.4	110.8 ± 4.0	−34.1 ± 5.2
Gilt-MNC-2	10	0.3	148.6 ± 7.9	−35.2 ± 4.3
Gilt-MNC-3	10	0.4	176.1 ± 14.4	−38.2 ± 3.5

## Data Availability

Data available on request.

## Supplementary Materials

The following supporting information can be downloaded at: https://www.mdpi.com/article/10.3390/polym16020225/s1, Figure S1: Representative RP-HPLC chromatogram of GLT solutions at varying concentrations; Figure S2: Leakage of GLT from Gilt-MNC incubated in 10 mM PBS (pH 7.4) without or with 10% FBS; Figure S3: Flow cytometry histograms showing the accumulation of DyLight488-labeled Gilt-MNC in 293T and HBMS cells following the treatment for 4 h with or without excess HA or dextran. Figure S4: Cytotoxicity of native EGCG, HA-EGCG, and Gilt-MNC-1 against MOLM-14 and MV-4-11 cell lines at varying EGCG unit concentrations; Figure S5: Median-effect plots derived from the cytotoxicity of free GLT, HA-EGCG, or their combination (Gilt-MNC) against MOLM-14 and MV-4-11 cell lines.
