# Supplementary material for: Potentiating Gilteritinib Efficacy Using Nanocomplexation with a Hyaluronic Acid–Epigallocatechin Gallate Conjugate
Source: Polymers (Basel). 2024 Jan 12;16(2):225. doi: 10.3390/polym16020225 (PMC10818662; doi:10.3390/polym16020225)
Supplement: Supplementary file 1 [file polymers-16-00225-s001.zip › polymers-2798433-supplementary.pdf]

*Supporting Information for*

# Potentiating Gilteritinib Efficacy by Nanocomplexation with Hyaluronic Acid-Epigallocatechin Gallate Conjugate

**Ki Hyun Bae [1,2], Fritz Lai [3], Qingfeng Chen [3,4], and Motoichi Kurisawa [2,5,*]**

[1] Bioprocessing Technology Institute (BTI), Agency for Science, Technology and Research (A*STAR), 20 Biopolis Way, Centros #06-01, Singapore 138668, Republic of Singapore; khbae@bti.a-star.edu.sg (K.H.B.)

[2] Institute of Bioengineering and Bioimaging (IBB), Agency for Science, Technology and Research (A*STAR), 31 Biopolis Way, The Nanos #08-01, Singapore 138669, Republic of Singapore

[3] Institute of Molecular and Cell Biology (IMCB), Agency for Science, Technology and Research (A*STAR), 61 Biopolis Drive, The Proteos, Singapore 138673, Republic of Singapore; sclai@imcb.a-star.edu.sg (F.L.); qchen@imcb.a-star.edu.sg (Q.C.)

[4] Key Laboratory for Major Obstetric Diseases of Guangdong Province, The Third Affiliated Hospital of Guangzhou Medical University, Guangzhou, China

[5] School of Materials Science, Japan Advanced Institute of Science and Technology, 1-1 Asahidai, Nomi, Ishikawa 923-1292, Japan; kurisawa@jaist.ac.jp (M.K.)

*Correspondence: kurisawa@jaist.ac.jp

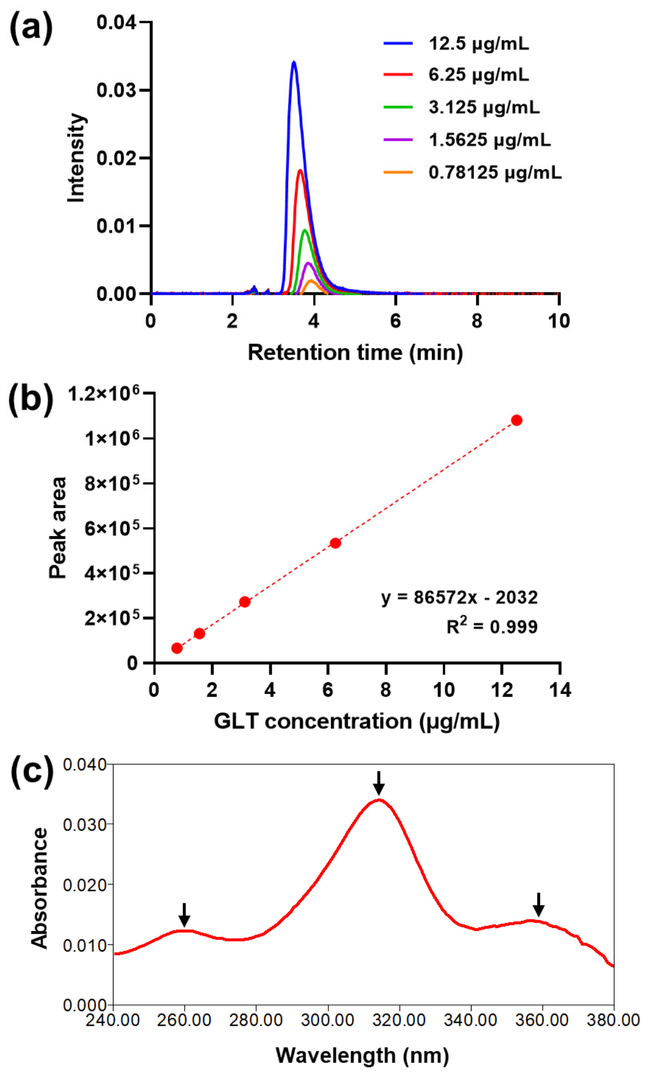


**Figure S1**. (**a**) Representative RP-HPLC chromatogram of GLT solutions at varying concentrations. (**b**) Calibration curves obtained with the average peak area of 5 different GLT concentrations. (**c**) UV absorption spectrum of GLT solution at a concentration of 12.5 µg/mL. The arrows indicate the characteristic peaks of GLT at 260, 314 and 358 nm.

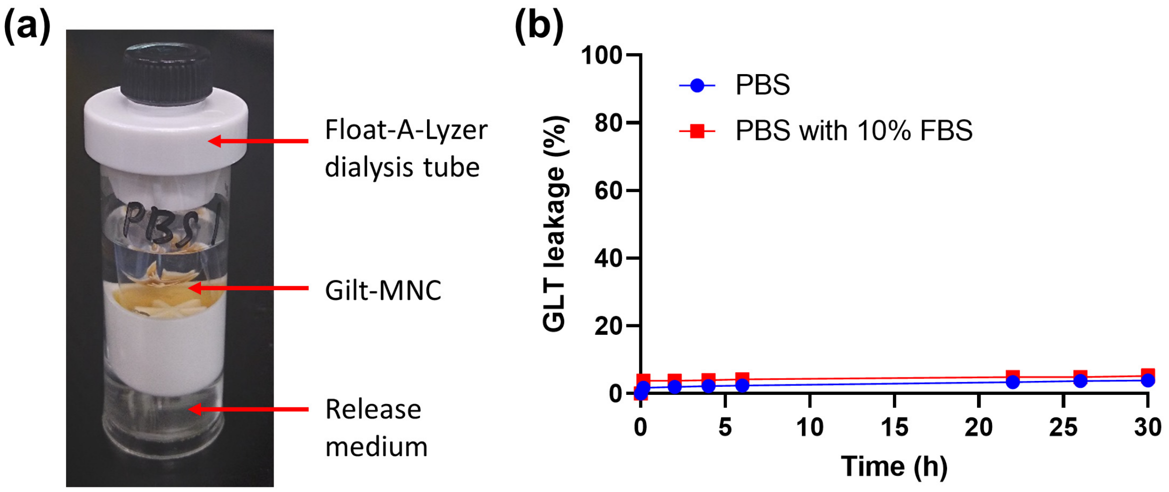


**Figure S2**. (**a**) Photograph showing the experimental set-up for GLT release study. (**b**) Leakage of GLT from Gilt-MNC incubated in 10 mM PBS (pH 7.4) without or with 10% FBS. Mean ± SD ($n$ = 4).

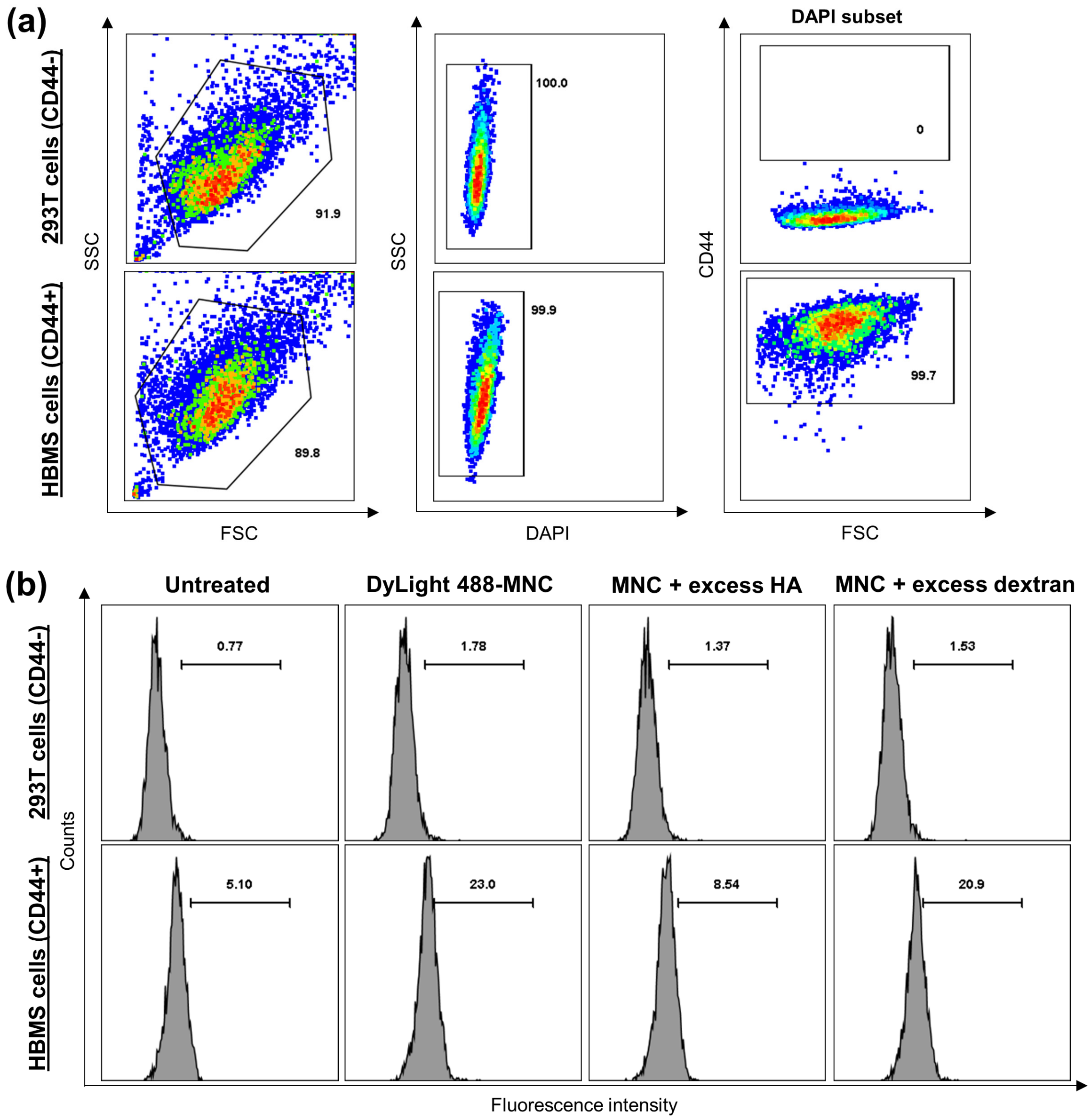


**Figure S3**. (**a**) Evaluation of CD44 expression on 293T and HBMS cells. (**b**) Flow cytometry histograms showing the accumulation of DyLight488-labeled Gilt-MNC in 293T and HBMS cells following the treatment for 4 h with or without excess HA or dextran.

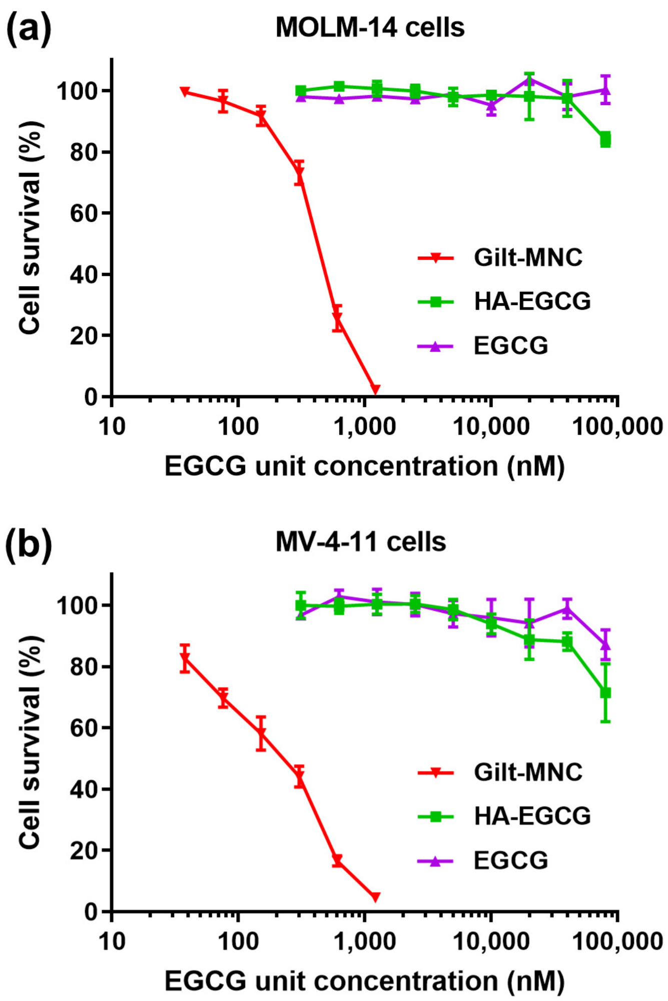


**Figure S4**. Cytotoxicity of native EGCG, HA-EGCG and Gilt-MNC-1 against (**a**) MOLM-14 and (**b**) MV-4-11 cell lines at varying EGCG unit concentrations. Mean ± SD ($n$ = 3).

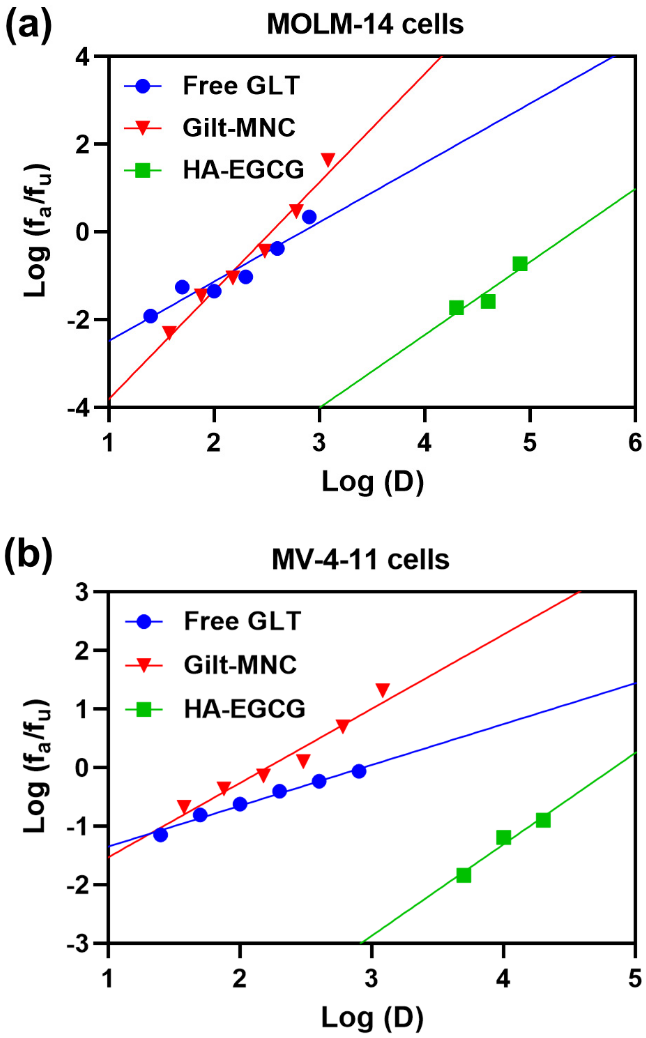


**Figure S5**. Median-effect plots derived from the cytotoxicity of free GLT, HA-EGCG or their combination (Gilt-MNC) against (**a**) MOLM-14 and (**b**) MV-4-11 cell lines.
